# Integrating Research-Supported Coaching Practices Into Secondary Teachers’ Team Meetings: Early Indications of Potential to Impact Collaborations, Classroom Interactions, and Student Engagement

**DOI:** 10.3389/feduc.2022.883226

**Published:** 2022-05-31

**Authors:** Megan Stuhlman, Amori Yee Mikami, Tara Hofkens, Joseph Allen, Robert Pianta, Sophie Smit

**Affiliations:** 1 Center for Advanced Study of Teaching and Learning, School of Education and Human Development, University of Virginia, Charlottesville, VA, United States,; 2 Peer Relationships in Childhood Laboratory, Department of Psychology, University of British Columbia, Vancouver, BC, Canada,; 3 Adolescent Research Lab, Department of Psychology, School of Arts and Sciences, University of Virginia, Charlottesville, VA, United States

**Keywords:** teacher collaboration, teacher professional development, secondary teachers, professional learning communities, classroom interactions, student engagement

## Abstract

The My Teaching Partner-Secondary (MTPS) program demonstrated improvements in classroom interactions and student outcomes in secondary schools using one-on-one coaching between study staff and teachers. Despite promising results, the time, cost, and oversight from a university research team may pose barriers to adoption of coaching programs like MTPS at scale. The My Teaching Team (MTT) project sought to translate key ingredients from MTPS into existing professional development contexts that are already built into many middle and high school educators’ weekly schedules: co-planning or professional learning community meetings. Six teams of secondary teachers (*N* = 30 teachers) participated in a pilot test of the usability of MTT materials across 5 months in one school year. Three teams elected to use MTT materials, and three elected to be a comparison group who continued their typical practices. Teams adopting MTT materials were observed to do so with good implementation integrity, and reported satisfaction with the intervention. Compared to typical practice teams, those using MTT were observed to spend more meeting time discussing teaching practice and less time discussing logistics/mechanics, and engaged in more video sharing and feedback to team members in the MTT sessions that explicitly encouraged this. The number of MTT meetings completed by a team, as well as spending more time discussing teaching practices and video sharing (but not feedback provided) during team meetings, predicted students’ self-reports of greater engagement and observations of higher levels of emotional support provided in the classroom. Implications for translating empirically supported interventions from the lab to real-world school settings are discussed.

## INTRODUCTION

Engaging and motivating interpersonal interactions with classroom teachers are key to optimizing learning outcomes among adolescents (Benner et al., 2008; Allen et al., 2013; Wang et al., 2020). My Teaching Partner-Secondary (MTPS) is a program involving one-on-one coaching between study staff and teachers, focused on improving such interactions in secondary classrooms, which has shown promising impacts on students’ academic achievement and engagement (Allen et al., 2011, 2015; Gregory et al., 2014). One-on-one coaching clearly offers many advantages to educators with access to this type of support, but is also resource- and time-intensive. The current study tested the feasibility and preliminary efficacy of My Teaching Team (MTT), an adaptation of MTPS that sought to maintain the core components of this successful intervention, but varied the delivery format to determine if a group-based, teacher-led model has the potential to demonstrate some of the same benefits.

## FOCUSING ON TEACHER-STUDENT INTERACTIONS TO IMPROVE ADOLESCENTS’ LEARNING OUTCOMES

Developmental research indicates that adolescents’ experiences of relational support, autonomy and competence, and understanding of the relevance of academic content all promote their learning (e.g., Deci and Ryan, 2000; Davis, 2013; Ruzek et al., 2016). Specifically, teacher efforts to provide relational supports by connecting with students and allowing students to feel known can enhance adolescents’ motivation in school and emotional functioning outside of school (Skinner et al., 1998; Pianta, 2011). In terms of autonomy and competence, adolescents are engaged by challenges that promote their sense of self-efficacy, blending self-direction with appropriate structure and support (Vansteenkiste et al., 2004; Sierens et al., 2009). Finally, although youth attach importance to the broader relevance of what occurs in the classroom (Bronfenbrenner, 1979), too often the connections between the secondary school curriculum and out-of-school contexts are not made explicit to students (Gainsburg, 2008).

The MTPS program was designed to help secondary school teachers incorporate these principals into their classroom interactions. Teachers engaged in one-on-one, biweekly meetings over a school year with a dedicated coach who encouraged teachers to video record their own classroom practices. These videos (as well as video exemplars of other teachers’ classrooms) were subsequently reviewed by both the teachers themselves and their coaches (Downer et al., 2011; Knight et al., 2012). Coaches provided feedback to teachers based in an empirically validated system for observing, coding, and ultimately changing teacher-student interactions to align with the developmental needs of adolescents: The Classroom Assessment Scoring System—Secondary (CLASS-S, Pianta et al., 2008a). MTPS coaching focused on enhancing emotional supports *via* making personal connections, being responsive to students’ needs, and considering students’ perspectives; organizing classroom activities to maximize engagement; and offering instructional supports that encourage dialogue, analysis, and metacognition. In MTPS, the coaches are study staff members, who are trained and supervised by the research team.

Results from several randomized trials (Allen et al., 2011, 2015) document improvements in quality of teacher-student interactions by the end of the intervention, which mediated better student achievement with new groups of students, after coaching ceased. Gains from exposure to an MTPS-trained teacher were equivalent to moving the average student from the 50th to the 59th percentile in achievement test scores (Allen et al., 2011, 2015). MTPS also led to sustained reductions in student disciplinary referrals and in racial disparities in discipline practices (Gregory et al., 2016). Similar positive results were found using the MTP coaching program with early childhood teachers (Pianta et al., 2008b).

My Teaching Partner-Secondary efficacy might be driven by its focus on observing and identifying effective teacher-student interactions in videos, using the lens of the CLASS-S to focus on interactions that provide relational support, foster student autonomy, and emphasize content relevance. Indeed, teachers who spent more time reviewing and analyzing their own videos with prompts from their coach demonstrated greater changes in their classroom practice (Pianta et al., 2014a). Other studies indicate that the ability to notice effective interactions in video exemplars can be developed through practice (Hamre et al., 2012), and teachers who get better at this skill over time improve their classroom instruction (Pianta et al., 2014b).

## DEVELOPING A MORE SCALABLE INTERVENTION: MY TEACHING TEAM

Although the MTPS program garnered strong empirical support in research trials, schools wanting to adopt MTPS more broadly may face challenges to making these effective supports available at scale. These challenges include schools having to change their existing professional development structure to accommodate coaching, teachers finding time outside other responsibilities to engage in coaching, monetary costs for hiring external coaches or training and supervising local coaches to ensure fidelity to the model, and acquiring enough trained coaches to implement the program. Relatedly, because the coaching occurs one-on-one betwen a study staff member and a teacher, MTPS may be less likely to become embedded in school culture, which may in term limit the potential for ripple effects into the school and the sustainability of the intervention.

We therefore sought to embed the active ingredients of MTPS into an alternative delivery format that aligns with professional development opportunities that already exist in many schools. Most teachers across the United States (75–80%) participate in regular, collaborative team meetings on issues of instruction (Wei et al., 2010; Garcia and Weiss, 2017). This teaching team format has the potential to support positive teaching culture and student learning (Vescio et al., 2008; Campbell et al., 2016), particularly when meetings adopt protocols that encourage collaborative practices including sharing actionable ideas, self-reflecting, offering feedback to others, and engaging in instruction-focused action planning (Vescio et al., 2008; Met Life, 2010; Wei et al., 2010; Mindich and Lieberman, 2012; Dogan and Adams, 2018). Notably, such collaborative practices do not characterize all team meetings (Little, 2003; Wei et al., 2010; Johnson et al., 2018).

**FIGURE 1 |:**
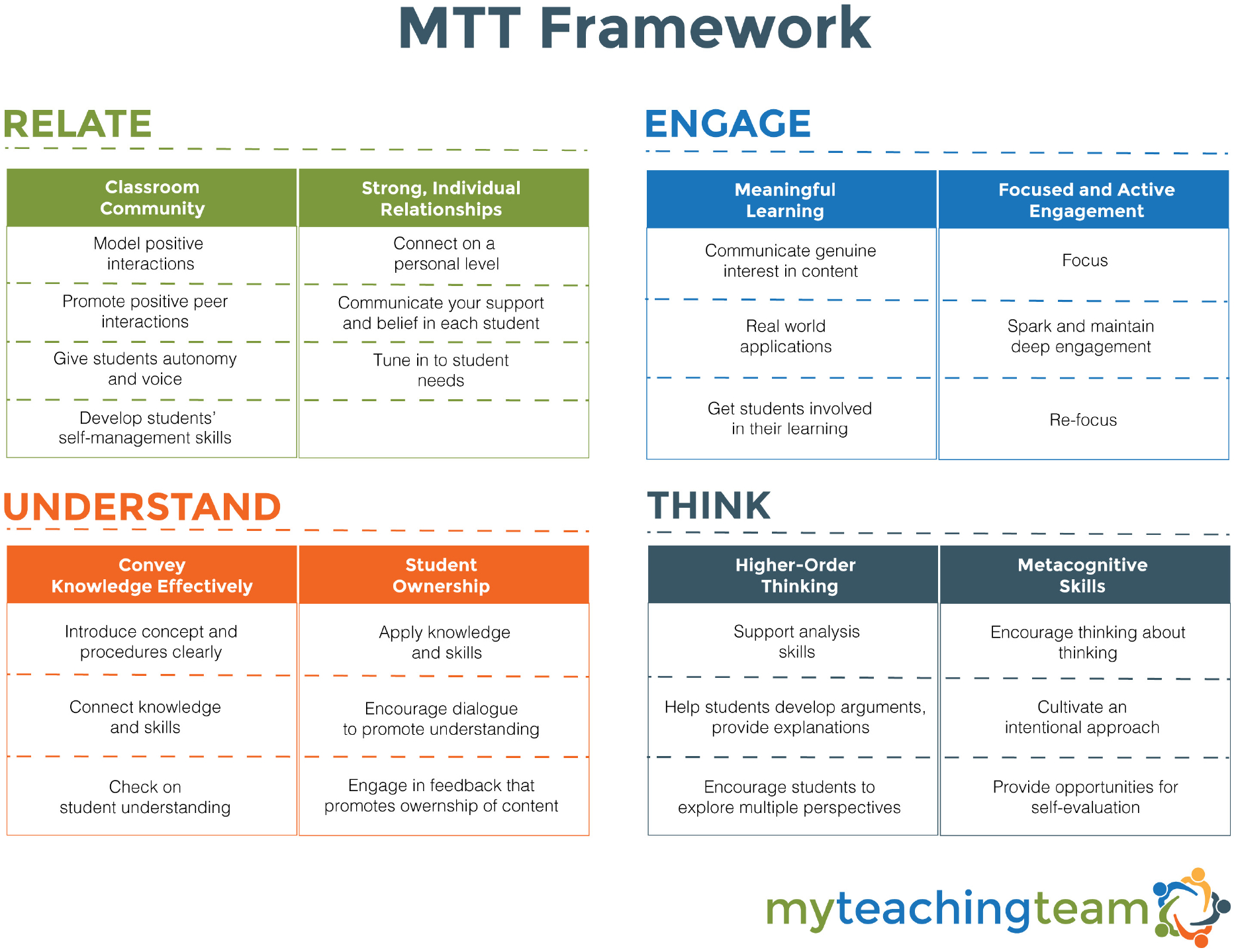
MTT framework topics.

Guided by existing research on teaching teams, as well as on what core ingredients made MTPS successful, we created the MTT intervention. A side-by-side comparison of MTPS and MTT logistic practices and components is provided in Table 1. MTT provides teaching teams with scaffolded materials that guide them through a sequence of steps to create a peer learning context that shares processes with video-based coaching protocols. Key MTPS components which were adopted by MTT included planning for and recording one’s classroom practices, analyzing one’s own (and peers’) videos, and focusing on empirically supported aspects of teacher-adolescent interactions represented in the CLASS-S framework. MTT builds on findings that specific CLASS-S dimensions used in MTPS coaching showed outsized impact on student outcomes (Allen et al., 2013; Gregory et al., 2014, 2016) by highlighting practices such as use of varied learning modalities and formats that encouraged active student participation, support for problem solving, perspective taking and prediction, and awareness of and responsiveness to students’ academic and emotional needs (see the MTT Framework; Figure 1). Like MTPS, the MTT Framework focuses on encouraging teacher behaviors that have the end goal of cultivating students’ feelings of relatedness, as well as their engagement, understanding of the relevance of the content covered, and thinking skills including analysis, perspective taking, and metacognition. To encourage generalizability, MTT Framework topics also mirror language used by collaborating school divisions in their mission statements or strategic plans.

The structure of MTT sessions encourages teachers to engage in collaborative practices that mirror the one-on-one coaching in MTPS. These include self-reflecting on successes and challenges in interactions with students, providing supportive and actionable feedback to peers, planning to expand successful practices or engage in new practices, and recording implementation of those plans for later review and analysis using guided prompt questions. After teachers have had the opportunity to try their plans in class, sessions focus on sharing video clips of their plan implementation with their peers and asking for specific feedback.

Unlike MTPS, MTT uses a group format for coaching and existing school-based personnel as group facilitators. This reduces the cost of coaching as well as increases sensitivity to specific school-level constraints, resources, and needs. Empowering teachers to lead their own discussions builds capacity within schools and holds promise for fostering teachers’ sense of professionalism, ownership, and collegiality (Parlar et al., 2017) that can translate in to sustainable, system-level shifts in teaching practices. The MTT approach also might establish enduring support systems between teachers (Vescio et al., 2008). A challenge, however, of using school-based personnel as facilitators (compared to study staff members) is providing sufficient support for implementation integrity. An additional challenge is fitting each of the targeted intervention topics into the time allotted for team meetings in partnering school divisions. As a result, the frequency of video recording and sharing of video is reduced in the MTT intervention in comparison with the MTPS intervention.

## RESEARCH QUESTIONS

The present study explored the feasibility and impact of translating elements of the effective MTPS program into a teacher-led team meeting format, MTT. In this pilot study, three teaching teams of secondary school teachers elected to use MTT materials, and three teams served as a comparison group who continued their typical practices.

### Research Question 1: Is It Feasible for Teams to Use My Teaching Team Materials and Does It Change Their Use of Meeting Time?

Among the teams who adopted MTT, we examined time teachers spent using MTT materials and integrity of implementation. We also compared MTT teams, relative to teams in the typical practice comparison group, in their use of research-supported collaborative practices and in what was discussed during meetings.

### Research Question 2: Do Teachers Perceive My Teaching Team Materials to Be Useful?

Among the teams who adopted MTT, we collected teachers’ reports of their experiences using MTT materials, and examined whether these perceptions differed based on teacher characteristics including education level, years of experience, motivational beliefs, and perceptions of school professional learning environment.

### Research Question 3: Is Higher Dosage of My Teaching Team Meetings Associated With Classroom Experiences?

Across all teams, we examined whether the number of MTT sessions completed related to observations and student self-reports of teacher-student interactions and student engagement.

### Research Question 4: Are Aspects of Meetings That Differentiated My Teaching Teams From Typical Practice Comparison Groups Teams Associated With Classroom Experiences?

We examined whether the discussion topics and collaborative practices that were more common in MTT team meetings related to observations and student self-reports of teacher-student interactions and student engagement.

## MATERIALS AND METHODS

### Participants

Participants were six pre-existing, discipline-based teacher teams (30 teachers) in two middle and two high schools within one suburban district in the southeastern United States during the 2019–2020 academic year. Three teams at three schools (19 teachers) elected to use MTT practices, and three teams in another school (11 teachers) elected to continue with their typical practices. All teams (MTT and typical practice comparison) agreed to allow the research team to collect data on their team interactions, classroom interactions, and student experiences (which included observations, survey completion, distributing and collecting consent forms from families and students, and giving class time for student survey completion).

Teams who elected to use MTT materials voted unanimously to engage in the research project. In addition to participating in data collection, this required all team members to attend orientation meetings, try a new structure and focus in team meetings, record interactions in their classroom, review those recordings and share segments with their teammates, and additionally required the team facilitator (1 per team) to attend monthly facilitator support sessions and lead their teams through the MTT process. These teams verbally communicated to study personnel that their existing goals were well aligned with the MTT goals of sharing practices and focusing on engaging students and they saw this as an opportunity to push their agenda forward with the help of the provided suite of resources.

Teams who elected to be a typical practice comparison group by continuing their existing team meeting practices but engaging in all data collection communicated to study personnel that they felt too overwhelmed by existing initiatives and demands on team meeting time to take on the extra tasks that MTT participation would involve (i.e., orientation, facilitator support meetings and using untested meeting practices). Nonetheless, they were invested in engaging in best practices in team meetings, their school was focused on increasing student engagement and relatedness, and they were interested in contributing to the study and in applying what was learned from the research in the future.

Although teams were not randomized into conditions, teachers in the MTT and comparison groups were overall similar in level of education, years of teaching experience, self-identified gender, and self-identified race/ethnicity (see Table 2). There were more teachers per team on average in the MTT group relative to the comparison group (6.33 vs. 3.66). This is likely due to the fact that while all teachers in teams adopting MTT needed to consent to participate in the full research project (given that we were asking them to change their typical practice), not all teachers in the comparison teams were required to participate in the research project. Therefore, a comparison team may have had more members than were reflected in our participant numbers. These team members would have attended meetings as usual but they would not have completed surveys, had their classrooms observed, or had their students surveyed.

A total of 224 students from enrolled teachers’ classrooms volunteered to provide study data (190 from classrooms of teachers in MTT teams and 34 from classrooms of teachers in comparison teams; significantly more consenting students in the MTT teams [*t*(28) = 3.11, *p* = 0.004]). An average of 10 students per MTT classroom, and three students per comparison group classroom, participated by completing surveys about their classroom experiences. Although data on number of students enrolled in each class was not collected for all classrooms, it is very likely that differences in numbers of consented students were related to higher investment in distributing and collecting consent forms by MTT teachers in comparison with comparison group teachers, rather than reflecting systematically larger class sizes for MTT teachers.

### Procedure

In the school district from which participants were recruited, pre-existing discipline-based teaching teams (each with a designated team lead) met for at least 45 min weekly. We began recruitment by providing study information to secondary school principals and department specialists. Among those expressing interest, information was shared with teaching team leads, who then shared study details with all members in their teams. Teams who decided to enroll in the research study either agreed to adopt MTT materials or to be a typical practice comparison group.

#### My Teaching Team Condition Activities

In October, teachers on MTT teams attended 2.5 h of orientation (overview of study procedures, MTT content, and MTT process) with study personnel. They were given consent/assent forms to distribute to students/guardians in a “typical class” chosen by the teacher, and completed surveys about their teaching beliefs and practices. Around the end of the first semester, after teachers had begun using MTT materials (mean number of completed meetings with MTT content = 1.78; range = 1–3), teachers permitted study staff to video-record one period of the typical class they had selected. In the second semester (mean number of completed meetings with MTT content = 6.44; range = 5–9), consented students completed surveys about their experiences.

Existing lead teachers served as their team’s MTT facilitator, guiding teams through MTT sessions and video-recording these meetings. To support implementation integrity, a defined agenda and multi-media materials were provided for each MTT session, and all teachers received handbooks that explained the MTT topics. Facilitators also met with a study staff member 1 h per month to discuss implementation and preview upcoming session materials.

Figure 1 displays the topics covered in MTT sessions over the course of a school year. Teams use a three-step process for each topic. Step 1 entails an initial “Focus/Plan” meeting that contains an orientation to the topic, including example practices and narratives provided by secondary school teachers, and discussion prompts to help team members share current practices. Video examples of teachers implementing practices related to the MTT topic are included, with structured analysis prompts for teams to discuss as they watch the videos. After these discussions, teachers use step-by-step planning forms to create individualized action plans for incorporating strategies related to the MTT topic into upcoming classroom interactions. Step 2, “Practice,” occurs back in their classrooms. Teachers record themselves trying their planned strategy and analyze their recording using provided reflection prompts. They then select specific short clips of their video that they wish to bring to their team for feedback. Step 3, “Reflect/Share,” happens in the next meeting. Teachers share reflections and self-selected video clips illustrating successes and challenges related to strategy implementation, and receive peer feedback and support. Teams repeat this process for each MTT topic.

#### Typical Practice Comparison Condition Activities

Teachers in comparison teams attended a 45-min orientation where research activities were explained, student/guardian consent/assent forms were provided, and teachers completed the same surveys about their teaching as MTT teachers. Teachers in the comparison group permitted project staff to video-record one period of a “typical class,” and consented students completed surveys about their experiences in the same timeframes as occurred for MTT teachers. Each team leader was asked to video-record one team meeting per quarter.

### Measures

#### Feasibility and Use of My Teaching Team Materials

In order to address Research Question 1 (feasibility of implementing MTT and impacts on use of meeting time), MTT teams were asked to submit videos of their MTT meetings; 78% of such sessions were successfully video recorded (six were missing due to technical difficulties). Typical practice comparison teams submitted one team meeting per quarter for video coding.

Videos were double coded for indicators of implementation integrity, discussion topics, and research-supported collaborative practices; see descriptions below. The coders were research assistants who met every other week to discuss codes and minimize drift. Intraclass correlation coefficients (ICC, Shrout and Fleiss, 1979) between coders were calculated to indicate inter-rater reliability, and those with ICC > 0.60 were retained. At weekly meetings, codes were reviewed, with discrepancies discussed and resolved. Codes used in analyses reflect team consensus.

##### My Teaching Team Dosage

Dosage is operationalized as the number of MTT sessions held for each team and was assessed *via* facilitators’ reports and verified with dated video-recordings of submitted meetings. MTT teachers also reported the amount of time spent on MTT activities outside of meetings (i.e., reviewing their video recording or planning).

##### My Teaching Team Implementation Integrity

In MTT teams only, coders scored each session video for adherence to MTT program content (ICC = 0.749). Each scored item corresponded with a specific agenda item provided to facilitators (0 = *the agenda item was not observed to be covered*, 1 = *the item was covered incompletely or with some lack of integrity*, and 2 = *the agenda item was covered with complete integrity*).

##### Team Meeting Discussion Topics

Both MTT and typical practice comparison group meetings were coded for the amount of meeting time teachers spent discussing six different topics. Coded topics included: *teaching practice*, *challenging student behaviors*, *challenging technology systems*, *challenging school policies*, *logistics*/*mechanics* (such as when a test will be scheduled, locations of materials, timing of school events), and *other topics* unrelated to teaching. The challenges and the other topics codes came from pilot findings on their prevalence in team meetings. For each session, time spent on each topic was coded on a four-point scale (0 = *no time*, 1 = *a brief moment*, 2 = *significant time but less than half the meeting*, 3 = *significant time and more than half the meeting*). ICCs for these codes ranged from 0.812 to 0.879.

##### Research-Supported Collaborative Practices

Seven practices highlighted by previous research (Mindich and Lieberman, 2012; Dogan and Adams, 2018) as indicative of effective teaching team collaborations were coded in MTT and comparison group meetings. Five of these practices were explicitly embedded in the MTT agendas: *shared actionable ideas about teaching*, *self-reflected on practices*, *reviewed video of team members’ teaching*, *provided feedback to peers*, and *made plans for implementing effective teaching practices*. The remaining two practices, *discussed student data* and *discussed other professional development experiences*, were not part of the MTT intervention, and were included to determine the extent to which MTT might pose an opportunity cost by reducing discussions in these areas in comparison to typical practice.

For each session, the amount that the team engaged in each research-supported collaborative practice was coded on a three-point scale (0 = *did not occur*, 1 = *cursory occurrence*, 2 = *substantive occurrence*). ICCs for these codes ranged from0.613 to 0.758, with the exceptions of shared actionable ideas (always occurred) and discussed other professional development (never occurred).

#### Teacher Perceived Utility of My Teaching Team

In order to address Research Question 2 (teacher perceptions of MTT usefulness and predictors of these perceptions), teachers in the MTT group completed several self-report questionnaires.

##### Satisfaction With My Teaching Team Materials

Following each MTT session, teachers were asked rate (1) whether they felt the meeting was worth their time, and (2) whether they would change teaching practices as a result of the meeting, on a 1–5 Likert scale (1 = *strongly disagree*; 5 = *strongly agree*). These two items were correlated at *r* = 0.67 and aggregated to create an overall score.

##### Professional Learning Environment

As a potential predictor of satisfaction with MTT materials, teacher perceptions of the professional learning environment at their schools were assessed with the Shared Personal Practice and the Supportive Conditions—Relationships subscales of the Professional Learning Community Assessment (PLCA; Olivier et al., 2010). These subscales consist of 12 items rated on a 4-point scale, assessing teacher collaboration (e.g., “Staff members informally share ideas and suggestions for improving student learning”) and supportive relationships (e.g., “A culture of trust and respect exists for taking risks”) in the school environment. Because the 12 PLCA items had an alpha of 0.93 in our sample, they were combined into one scale for analyses.

##### Growth Mindset

As a second potential predictor of satisfaction with MTT materials, the Mindset about Intelligence scale (Dweck et al., 1995) was given to measure how much teachers viewed intelligence as fixed. The four items (e.g., “To be honest, you cannot really change how intelligent you are”) were rated on a 5-point Likert-type scale (1 = *strongly disagree* to 5 = *strongly agree*) and had high internal consistency in our sample (α = 0.97). We also adapted this scale to include four additional items that asses teacher beliefs about the malleability of teaching ability (e.g., “Teaching ability is a skill that you either have or you do not”), which also showed high internal consistency in our sample (α = 0.82).

#### My Teaching Team Dosage and Practices as Associated With Classroom Experiences

Research Question 3 and Research Question 4 (how MTT dosage and use of the practices that MTT promoted related to teachers’ classroom interactions with their students and student engagement) were assessed using classroom observations and student self-report questionnaires.

##### Classroom Observations of Interactions and Student Engagement

We coded classroom videos using the CLASS- S (Pianta et al., 2008a). The CLASS-S scales that were aligned with the MTT intervention were used for analytic purposes and consist of eight, seven-point rating scales in two domains assessing teacher-student interactions (Emotional Support and Instructional Support), as well as a Student Engagement scale. CLASS-S scores have been found to predict academic achievement outcomes over a school year (Allen et al., 2011, 2013).

One approximately 45-min class period was recorded for 29 participating teachers (one classroom was not recorded due to the teacher being on leave during the recording window). This recording was divided into two (*n* = 6 classrooms) or three (*n* = 23 classrooms) 15–20-min segments for coding purposes. Segment codes were averaged, resulting in one data point per classroom for each CLASS-S domain. Coders were certified reliable on the CLASS-S (assigning 80% or more codes within one point of master coders on five test recordings), and the average ICC of double-coded segments in the present study (19% of segments) was 0.70. Reliability coefficients for variables used as outcomes were in the “fair” to “good” range (Cicchetti and Sparrow, 1981): Emotional Support ICC = 0.71, Instructional Support ICC = 0.87, Student Engagement ICC = 0.51.

##### Student Self-Reported Engagement

We assessed students’ self-reports of their engagement in class using scales from Wang and colleagues (2020) on a 5-point metric from “1 = *not at all like me*” to “5 = *very like me*.” Cognitive engagement included six items about students’ use of deep learning strategies and self-regulated learning (e.g., “I try to connect what I am learning to things I have learned before”). Behavioral engagement contained seven items about investment and involvement in classroom activities (e.g., “I keep trying even if something is hard”). Emotional engagement included six items about students’ value of and positive and negative reactions to classroom learning and activities (e.g., “I look forward to class”). All demonstrated acceptable to excellent internal consistency in our sample (cognitive: α = 0.65; behavioral: α = 0.79; emotional: α = 0.91).

##### Student Self-Reported Classroom Interactions

Students completed a revised version of the *Classroom Assessment Scoring System Student Report* (Downer et al., 2015), which includes 24, five-point Likert scale items that tap into students’ perspectives of the CLASS domains utilized in the present study (Emotional Support and Instructional Support). Some items were revised to fit the secondary school context (e.g., “My teacher helps me when I need help” and “My teacher encourages me to share my ideas in class.”). These adapted items have been validated with secondary students; individual- and classroom-level student reports were associated with achievement and disciplinary referrals (Downer et al., 2015; Wang et al., 2016, 2020). The scale exhibits acceptable to very good internal consistency in this sample (Emotional Support: α = 0.77; Instructional Support: α = 0.85).

### Statistical Analyses

Data were analyzed using Statistical Packages for the Social Sciences (SPSS) 28.0 and Stata 15.1. For Research Question 1, we compared research supported collaborative practices and discussion topics in MTT sessions to those in typical practice comparison group sessions using Mann Whitney *U* tests, given the small sample size in each group and because our data are on an ordinal scale and non-normally distributed. For Research Questions 2–4, we used multiple linear regression, which is appropriate for a small sample with outcomes containing the level of variance demonstrated in our study (Jenkins and Quintana-Ascencio, 2020). We ran separate models for each outcome and included teacher gender and race/ethnicity in regressions predicting student-reported outcomes (Research Questions 2 and 4). Teacher gender and race were covaried in these models because they were of sufficiently high frequency to include, did not cause issues of multicollinearity (determined by examining the variance inflation factor), and the r-squared of the model was improved by including them (indicating that they contribute explanatory value). Due to the small sample size, we did not include covariates in regressions predicting teacher-reported outcomes (Research Question 3). For each analysis, we examined assumptions required for linear regression, including assessing the normality of the distribution of residuals (density and P-P plots, symmetry of the distribution across inter-quartile ranges, and the Shapiro-Wilk W tests for normality), homogeneity of variance of residuals (Cameron & Trivedi’s decomposition test), and non-collinearity among predictor variables (variance inflation factors).

## RESULTS

### Descriptive Statistics

Descriptive statistics of key study variables are in Tables 2–4. Comparisons between MTT and typical practice comparison group teams are in the tables, and presented in the results below.

### Research Question 1: My Teaching Team Feasibility and Impact on Use of Meeting Time

#### My Teaching Team Dosage

My Teaching Teams began using MTT materials in late October 2019 and were scheduled to meet approximately twice monthly until late May 2020. In March of 2020, however, all in-person instruction in the participating school district ceased due to the global pandemic, and so did our research activities. Up until March 2020, MTT teams’ meeting schedules closely approximated the recommended frequency (mean inter-session interval ranged from 2.14 to 1.78 weeks). Before instruction ceased, MTT teams held a total of 7–12 MTT sessions (mean = 9 per team); this included four to six Focus/Plan sessions, two to five recordings of classroom practice times per teacher, and three to six Reflect/Share sessions. This placed all teams roughly on target to complete 16 sessions had the school year continued as anticipated.

Between October and mid-March, MTT teachers reported spending, on average, 5 h 40 min of team meeting time participating in MTT sessions and 3 h 30 min analyzing and reflecting on their own classroom video footage. This translates to a mean time of 26 min/week spent on MTT-related activities.

#### My Teaching Team Implementation Integrity

Separate ratings were calculated for implementation integrity in Focus/Plan sessions (devoted to new topics and planning implementation) and Reflect/Share sessions (devoted to reflection and sharing of implementation). Results are summarized in Table 4 and indicate that overall, MTT teams showed high adherence to MTT agendas and materials, with some errors or omissions. Integrity in Reflect/Share sessions was slightly higher than in Focus/Plan sessions. The most consistently implemented elements were *discussion of the MTT topic* in Focus/Plan sessions and *sharing classroom videos* in Reflect/Share sessions.

#### Team Meeting Discussion Topics

Table 5 provides descriptive results for observed topics discussed during team meetings. Results indicate that MTT teams spent more meeting time discussing teaching practices and less meeting time discussing logistics/mechanics compared to comparison teams. There were no condition differences in time spent on other discussion topics.

#### Research-Supported Collaborative Practices

Table 5 also provides descriptive findings regarding the extent to which team meetings were characterized by seven research-supported collaborative practices. Occurrences of two of the measured practices were significantly different in MTT meetings (Reflect/Share sessions only) vs. typical practice comparison group meetings. Reflect/Share MTT meetings were more likely to include teachers *reviewing video of team members’ teaching* and *providing feedback to peers* relative to comparison group sessions. MTT meetings were not rated significantly differently from comparison group meetings in self-reflection on practices, making plans to implement effective teaching practices, or discussing student data. All observed team meetings included sharing of actionable ideas about teaching and no observed team meetings included discussion of other professional development experiences.

### Research Question 2: Teacher Perceived Utility of My Teaching Team Sessions

Overall, teachers in MTT teams reported high perceptions of the utility of MTT sessions, with average scores of 4.47 (SD = 0.38) on a 5-point scale ranging from strongly disagree to strongly agree (see Table 2).

According to density and P-P plots, examining the symmetry of the distribution across inter-quartile ranges, and the Shapiro-Wilk W tests for normality, there were no severe outliers and the residuals were normally distributed in the models that assessed whether perceived utility of MTT differed based on teacher characteristics. According to Cameron and Trivedi’s decomposition test, there was sufficient homogeneity of variance among the residuals. Finally, variance inflation factors indicated that there was not an issue with multi-collinearity among the predictor variables. Compared to teachers with a master’s degree, those with a bachelors’ degree reported higher perceptions of MTT utility [β = −0.70 (0.21), *p* = 0.03]. Also, the more positively teachers perceived the learning climate at the school, the more useful they perceived MTT to be [β = 0.53 (0.17), *p* = 0.03]. Neither years of experience teaching [β = −0.39 (0.01), *p* = 0.13], growth mindset about intelligence [β = −0.49 (0.14), *p* = 0.10], or growth mindset about teaching ability [β = −0.44 (0.71), *p* = 0.09], were significantly associated with perceptions of MTT utility (see Table 4).

### Research Question 3: Association of My Teaching Team Dosage With Classroom Experiences

For this research question, we used multiple linear regression to assess whether the number of MTT sessions in which teachers participated predicted classroom experiences, including observed teacher-student interactions and student engagement, and student self-reports of their engagement and their perception of classroom interactions. For teachers in the MTT condition, we recorded the number of MTT sessions they attended before outcome measure was administered; for comparison group teachers, the number of MTT sessions attended was always zero. We combined the MTT and comparison groups in this analysis to increase sample size.

Regarding assumptions for running linear regressions, we found that in the models with observed interactions, the residuals were normally distributed, there was sufficient homogeneity of variance among the residuals, and no issue was found with multi-collinearity. In the models with student-reports, the residuals were slightly positively skewed, which could contribute to the assessment of significance being less reliable. We present the results from the models without robust standard errors, but results were comparable when robust standard errors were used.

The number of MTT sessions that teachers attended before classroom observations occurred (range 0–3 sessions) predicted more observed emotional support in the classroom (β = 0.39, *p* = 0.04), but not instructional support or student engagement. The number of MTT sessions that teachers attended before the student surveys occurred (range 0–9 sessions) predicted student-report of more emotional (β = 0.18, *p* = 0.04) and behavioral engagement (β = 0.19, *p* = 0.03), and instructionally supportive interactions (β = 0.18, *p* = 0.03), but did not predict their cognitive engagement, or perception of emotionally supportive interactions in the classroom. Given the slight skew of the residual distribution in these models and given the relatively large *p*-value, we view these findings as preliminary and in need of replication.

### Research Question 4: Associations Between Practices That Differentiated My Teaching Team and Typical Practice Comparison Group Teams With Classroom Experiences

We conducted multiple linear regression analyses to assess whether the two discussion topics and two research-supported collaborative practices that differed between MTT and comparison group team meetings (see results for Research Question 1) predicted classroom experiences (see Tables 6, 7). Using the same assumption tests as in Research Questions 2 and 3, we found that in the models with observed interactions, the residuals were normally distributed, there was sufficient homogeneity of variance among the residuals, and there was not an issue with multi-collinearity. In the models with student-reports, the residuals were slightly positively skewed, which could contribute to the *p*-values being less reliable. We present the results from the models without robust standard errors, but results were comparable when robust standard errors were used.

Time teachers spent discussing teaching practice in meetings was positively associated with observed emotionally supportive interactions (β = 0.52, *p* = 0.005), instructionally supportive interactions (β = 0.39, *p* = 0.040), and student engagement (β = 0.44, *p* = 0.020). This variable was also positively associated with students’ reports of their behavioral engagement (β = 0.22, *p* = 0.017), emotional engagement (β = 0.19, *p* = 0.038), emotionally supportive interactions (β = 043, *p* < 0.001), and instructionally supportive interactions (β = 0.81, *p* < 0.001). In contrast, the time teachers spent discussing logistics/mechanics was negatively associated with observed emotionally supportive interactions (β = −0.56, *p* = 0.002), instructionally supportive interactions (β = −0.44, *p* = 0.020), and student engagement (β = −0.48, *p* = 0.009), as well as students’ reports of their behavioral engagement (β = −0.28, *p* = 0.001), emotional engagement (β = −0.25, *p* = 0.005), cognitive engagement (β = −0.19, *p* = 0.026), emotionally supportive interactions (β = −0.37, *p* < 0.001), and instructionally supportive interactions (β = −0.73, *p* < 0.001).

The extent to which teams shared video of team members’ classroom interactions in meetings positively predicted observed emotionally supportive interactions (β = 0.53, *p* = 0.004), instructionally supportive interactions (β = 0.42, *p* = 0.027), and student engagement (β = 0.46, *p* = 0.013), as well as students’ reports of their behavioral engagement (β = 0.22, *p* = 0.013), emotional engagement (β = 0.20, *p* = 0.028), emotionally supportive interactions (β = 0.41, *p* < 0.001), and instructionally supportive interactions (β = 0.75, *p* < 0.001). However, providing feedback to peers was negatively associated with student report of emotional support (β = −0.28, *p* = 0.001).

## DISCUSSION

This small pilot study is an early exploration of the extent to which key practices and positive impacts of a one-on-one coaching intervention lead by highly trained, university-based coaches (MTPS) could be retained when the format of the intervention was adapted to fit into a different professional development structure–team meetings led by the teachers themselves (MTT).

### Feasibility, Impact on Meetings, and Teacher Perceived Utility of My Teaching Team Materials

Teams were generally able to implement the MTT protocol as designed and regarded it as useful. Before the COVID-19 pandemic caused teaching teams to devote all of their attention to transitioning to remote instruction, teams covered approximately one MTT topic per month (holding two meetings and recording classroom interactions in between meetings), and did so with reasonable implementation integrity (particularly in Reflect/Share sessions). MTT activities took about half an hour weekly of teachers’ time, which does not seem prohibitive given the national average time teachers report having to collaborate with colleagues is close to 3 h per week (Wei et al., 2010). Using those parameters, MTT implementation would take approximately 20% of weekly allotted collaboration time.

The potential payoff for taking this time is that MTT teams spent a greater proportion of their meetings focused on teaching practices, and in the Reflect/Share sessions, they engaged in more sharing of videos of classroom interactions and provided more teaching focused feedback to one another. That the comparison group teams observed in this study spent less than half of their meetings discussing teaching practices and more than that focused on logistics/mechanics (such as when yearbooks would arrive or holiday celebrations would take place) resonates with previous findings that under typical circumstances, teacher teams rarely engage in transformational discussion about their teaching (McLaughlin and Talbert, 2006; Mindich and Lieberman, 2012) or in joint efforts to improve instruction and learning (Wei et al., 2010). Furthermore, previous findings indicate that teachers in typical collaborative groups rarely have opportunities to engage in peer observation and feedback (Met Life, 2010).

There did not appear to be a significant opportunity cost of using MTT, as MTT and comparison group teams discussed student data and other professional learning at similar rates. In fact, using existing frameworks for conceptualizing teacher collaboration, our data suggest that the comparison teams were functioning at the “storytelling and scanning for ideas” (Little, 1990) or the “coordination” or “cooperation” (Havnes, 2009) end of the continuum (as evidenced by their observed sharing of actionable ideas and by spending most of their meeting time discussing logistics/mechanics). MTT materials may support incorporation of practices consistent with the “joint work” (Little, 1990), “sharing” (Havnes, 2009), or “improving” stage, characterized by addressing challenges through brainstorming solutions, providing constructive feedback, and trying new implementation methods (see Nguyen and Ng, 2020).

Teacher perceptions of MTT utility were overall quite high. However, our findings that greater perceived utility was predicted by lower education level (bachelor’s vs. master’s degree) and a more supportive professional learning environment of the school, suggest directions for future research regarding what works for whom under what conditions (see Bryk et al., 2015). Capitalizing on the promise of teacher team meetings may entail situating our understanding of team practices both in the larger context of school culture, and in the more individual context of teachers’ personal characteristics.

### Promise of My Teaching Team Materials

Preliminary findings suggest that dosage of MTT, and more meeting time spent discussing teaching practice and reviewing video of team members’ teaching, are associated with multiple indicators of more positive classroom experiences, both observed and student-reported. Thus, this pilot study of MTT shows promise for supporting impactful teacher collaboration. In contrast, greater discussion of logistics/mechanics in team meetings (which was less common in teams using MTT) was consistently associated with less positive classroom outcomes. Contrary to hypotheses, teachers’ greater provision of feedback to peers in teacher team meetings was associated with less positive scores on one outcome variable: student self-reports of teacher emotional support. Perhaps our measure of providing feedback needs to be refined, such as by including specificity regarding whether the feedback is on-topic, strengths-focused, or actionable.

The indications that MTT dosage and use of discussion topics and research-supported collaborative practices encouraged by MTT were associated with more positive classroom experiences could have occurred because more skilled teachers are likely to volunteer to try new team meeting protocols that require sharing of their practices. However, it could also indicate positive effects of using MTT materials more frequently and engaging in the types of interactions central to MTPS and encouraged by the MTT protocol (video sharing, sharing challenges and successes, providing focused, actionable feedback, and making plans about how to engage in supportive teacher-student interactions). Specifically, these factors could contribute to more student engagement experienced at an emotional (“I look forward to class”) and behavioral level (“I keep trying even when the work gets hard”); greater student perceptions that teachers support their learning (e.g., “My teacher keeps working with me until I understand what we are doing”); and higher quality observed emotional support expressed in teacher-student interactions (i.e., being aware of and responsive to students’ academic and emotional needs). These indicators of effective classroom interactions and student experiences are well-established to relate to student learning outcomes (Allen et al., 2011; Wang et al., 2020), which suggests potential for MTT to eventually impact student achievement, as was found to occur in MTPS.

### Lessons Learned

Translating empirically supported treatments from university labs to real-world educational settings often requires paring down protocols to their most essential elements, offering autonomy and choice for users within well-articulated frameworks, and providing supports to practitioner-leaders that fit into their available time. The Reflect/Share MTT sessions (which were more streamlined and focused on teachers sharing their own practices) had somewhat higher implementation integrity and use of some research-supported collaborative practices in comparison with Focus/Plan sessions (which had more components and involved orienting to a new topic and articulating plans to incorporate practices related to that topic into existing lessons). In future refinements of MTT, we plan to reduce the number of components in team meetings and build in more choice points, such as allowing teams to decide which topics to cover in a given school year or how many meetings they devote to each topic. We also plan to create options for more time to be spent in Reflect/Share sessions.

Shifting from the one-on-one coaching format of MTPS (where coaches were highly trained study staff members) to the peer-coaching approach in MTT brought tradeoffs that could influence the impact of the intervention. For example, in order to achieve support from administrators and fit into the structure of the existing school system, MTT needed to reduce intervention intensity to bi-weekly group meetings and once monthly classroom video recording and sharing as opposed to the twice monthly video recording and review that characterized the more intensive individualized coaching in MTPS. MTT also removed intervention components that may be important drivers of change, such as teachers responding to coach-selected video segments and tailored written prompts, and receiving advice from a coach extensively trained and supervised by a research team. In MTT, all these elements were provided in a self- or peer-coaching format. Although peer coaching has demonstrated positive impacts on teaching efficacy (Papay et al., 2020), these impacts rely on at least one of the peers already being skilled in highly effective practices. MTT teachers’ baseline skills in teaching practice, making specific observations when viewing one’s own and others’ teaching, and providing feedback, were unknown. Prior work indicates that peer collaborations are more effective when collaborators have expertise/skill in effective teaching (Jackson and Bruegmann, 2009). In order to support or enhance these skills in our participants, MTT materials included “answers” to provided video analysis activities (to help ensure that conversations were on track). Further, MTT materials included specific prompts and structures for both giving and receiving feedback (i.e., prompting teachers sharing video to consider what they thought was both effective and ineffective about their video recorded interactions, how they knew those interactions were more/less effective, and what questions they wanted to ask their peers after they had viewed the video together; and prompting teachers giving feedback to listen carefully to the feedback request and then give feedback that was observationally based, specific, and relevant to/focused on the request). Because MTT was associated with more talk about teaching practice, sharing of practice (*via* video), and providing feedback as compared with comparison group meetings, these supports appear to have been successful in enhancing effective collaboration. However, future efforts may place more emphasis on ensuring that there are high-performing colleagues participating in each team (Jackson and Bruegmann, 2009; Papay et al., 2020).

Another key in shifting from expert one-on-one coaching to group-based peer coaching is group leadership. In the MTT intervention design, the teacher who serves in the position of team facilitator serves a key role in ensuring that discussions are in-depth and focused, that conversations about videos provide accurate and insightful analysis, that there is sufficient psychological safety for teachers to bring their concerns to the group, and that team members will receive both accurate and non-judgmental feedback and effective suggestions for enhancing practices from peers (a role served by the coach in MTPS). In this pilot study, team facilitators received monthly support from project staff that included staff reviewing video of each MTT team meeting and highlighting effective practices observed in meetings as well as offering suggestions for strategies to enhance MTT implementation. Future efforts to support high-impact team collaborations such as MTT should include provisions to provide practice-based supports for teachers serving in the role of facilitator or team lead to ensure that these leaders have the capacity to chart a positive course for team interactions and have the skill to recognize and give feedback on both more and less effective teaching practices.

An element added in the MTT delivery format that is not present in individual coaching protocols (such as MTPS) is the opportunity for teachers to receive support from multiple perspectives and from their peers. Notably, teachers may feel that their peers have more credible suggestions than do external coaches, as they share the same role, rather than being one step removed from the classroom. Relatedly, the group format of MTT adds an element of collective collaboration that may feel less prominent in individual coaching. This spirit of “joint work” (Little, 1990), where teachers are actively engaged in supporting the professional growth of their colleagues, may be key to enhancing teaching practices that in turn support student learning and development (Katz and Dack, 2014). Katz and Dack (2014) have reported that the key to effective professional development “is to create the conditions for generating new knowledge through a process that combines deep collaboration with evidence and inquiry.” (p. 36). The MTT intervention has endeavored to bring these three elements together through its collaborative format, its use of video as evidence of teaching practices (and the impact on students), and its prompts for active inquiry in each aspect of team meetings.

### Study Strengths and Limitations

A strength of this pilot study was its ambition to translate an intervention established in the lab (MTPS) into a more sustainable and feasible format for adoption by schools. Another strength was the multiple sources of data obtained, including teacher and student self-reports, and observational coding of team meetings and teacher-student interactions in the classroom.

Due to small numbers of participants, this pilot study has significant limitations in statistical power to detect effects and substantial restrictions to generalizability. Relatedly, we did not control for family-wise error rate across analyses, because of the pilot nature of our study. The lack of randomized assignment of teams to MTT or typical practice comparison conditions and the fact that all three comparison teams were in one school setting, whereas the MTT teams were distributed across three different schools, further prohibit causal conclusions related to observed differences in classroom experiences. The existing culture around professional learning in the comparison school may have differed from the culture in the three MTT schools, and there may have been important variability across the three MTT schools that could influence team practices, teacher-student interactions, and student engagement as well. However, we did collect data on teachers’ perceptions of the professional learning environment at their school in the fall of the school year prior to beginning the intervention and found that there were no school-level differences in teachers’ perceptions of the school learning environment (neither in shared professional practice nor supportive relationships subscales) or in teachers reports of work-related stress.

There were also substantial differences in numbers of students consenting to complete surveys between classrooms of teachers in the comparison group vs. in classrooms of teachers utilizing the MTT materials, which we attribute to differences in teacher enthusiasm for the research program.

In addition, both the MTT intervention itself and associated data collection were truncated due to the global COVID-19 pandemic. Analyses using number of sessions held was utilized as a method for enhancing our understanding of potential impacts, but it is incomplete. Further research is necessary to tease apart whether MTT truly enhanced team practices, teaching practices, and student experiences or whether teams who initially volunteered to try the practices were already more likely to engage in effective team practices and have better classroom interactions with students.

In light of all of these limitations, we consider all of these analyses to be preliminary and encourage replication.

## CONCLUSION

Despite these limitations, the pattern of preliminary results obtained suggests that MTT shows promise as a scalable, feasible way to embed some of the active ingredients of the successful MTPS coaching model into a teaching team meeting format, which upwards of 75% of teachers already use (Wei et al., 2010). The national staff development council has suggested that “a more systematic approach to support the productive use of common planning time might strengthen the continuous improvement cycle of professional development” (Wei et al., 2010, p. 20). Further development and refinement of the MTT intervention and evaluation of its impacts on teacher collaborative practices, classroom interactions, and student experiences could help determine if MTT offers an effective response to that call to action.

## Figures and Tables

**TABLE 1 |:** My Teaching Team compared with My Teaching Partner-Secondary.

	My Teaching Team	My Teaching Partner Secondary
**Goal of intervention**	To enhance teachers’ knowledge about, ability to identify, and implementation of effective interactive practices in their classroom, which in turn leads to better student experiences and outcomes.	
**Logistics of intervention**		
Format	Peer group of variable numbers of teachers (4–10)	1:1 meetings between an external coach and teacher
Time requirement	Intervention takes place during existing team meeting time created by schools.	Intervention takes place outside of existing professional learning structure, scheduled individually by teachers with their coaches.
Primary person responsible for content delivery	Participating teacher serving as group facilitator	External coach who is a study staff member
How is content determined	MTT Handbook given to group facilitator outlines topics and processes used in and between sessions.	MTPS Coaching Manual given to external coach outlines topics and process used in and between sessions.
How are teachers introduced to content	Group facilitator leads discussion about content, including showing video clips (provided by MTT) with analysis prompts.	Coach leads discussion about content, including asking teachers to view video clips (provided by MTPS) between coaching sessions.
Frequency of meetings	Suggested bi-monthly (one meeting for focus/plan components; the second meeting for reflect/share components)	Suggested bi-monthly (both meetings include focus/plan/reflect/share components)
**Intervention components**		
Are focus topics derived from empirically supported interactive practices?	Yes, focus topics based on CLASS dimensions that showed highest impact in MTPS studies	Yes, focus topics based on CLASS dimensions
Are focus topics integrated into discussion of strengths and challenges in current practices?	Yes, via standard discussion prompts	Yes, individualized based on coach discretion
Are action plans created?	Yes, in sessions, using provided planning forms with specific prompts	Yes, in sessions and emailed to teachers by coaches after sessions
Do teachers video record their implementation of their action plan?	Yes, independently	Yes, independently
Suggested frequency of classroom video recording	Once monthly	Twice monthly
Are teachers’ classroom video recordings shared for feedback?	Yes	Yes
Do teachers preview their own video recordings before sharing for feedback?	Yes	No
Who identifies portions of classroom video to focus on for feedback?	Teachers select their own clip	Coach selects clip
Is written feedback provided on classroom video?	No	Yes, by coach
Is verbal feedback provided on classroom video?	Yes, by peers in team meetings	Yes, by coaches in 1:1 coaching sessions

**TABLE 2 |:** Descriptive statistics on study participants.

Variable	MTT teams			Comparison teams			Total	Indep. *T*-test for equality of means	Pearson Chi-Square Test
Number of schools represented	3			1			4	n/a	
Number of teaching teams	3			3			6	n/a	
	Team 1	Team 2	Team 3	Team 4	Team 5	Team 6			
Number of teachers^1^	4	5	10	2	6	3	30	n/a	
Number of consenting students^2^	26	45	119	20	9	5	224	3.11**	
Mean years teaching experience	14.50 (13.2)	11.00 (7.17)	13.80 (11.22)	14.50 (2.12)	7.83 (4.26)	8.67 (6.51)	11.77 (8.79)	−1.91	
Teachers’ education level^3^	2.50 (1.00)	1.80 (.84)	2.30 (.95)	3.00 (1.4)	2.83 (.75)	3.33 (2.52)	2.43 (1.24)		7.55
Teachers’ self-identified race/ethnicity	100% White	100% White	20% Latinx 10% Multiple Identities 70% White	50% Black 50% White	17% Multiple Identities 83% White	100% White	3.3% Black 6.7% Latinx; 6.7% Multiple Identities; 83.3% White		3.04
Teacher female	50%	80%	80%	50%	50%	33.3%	63.3%		2.39

1 There are insufficient numbers of teams to examine if the number of teachers per team is statistically significantly different across the MTT and comparison groups.

2 Mean of 55% of enrolled students participated in classrooms where we have this information (*n* = 6; MTT only).

3 1 = BA; 2 = BA+; 3 = MA, 4 = MA+/EdSp.

** p = 0.01.

**TABLE 3 |:** Descriptive statistics of study variables.

	MTT teachers			Comparison teachers			ANOVA *F*-test
	*N*	Mean (SD)	Range	*N*	Mean (SD)	Range	
**Teacher perceptions**							
Teacher perceptions of school learning climate	19	3.07 (0.41)	2.42–4.00	11	3.07 (0.41)	2.41–4.00	0.30
Fixed mindset about intelligence	19	2.60 (0.55)	1.38–3.63	11	2.94 (0.77)	1.88–4.50	0.23
Fixed mindset about teaching ability	19	2.17 (0.76)	1.00–4.25	11	2.02 (0.90)	1.00–4.00	2.04
**Classroom experiences**							
Observation of interaction quality							
CLASS: Emotional support	18	4.88 (0.58)	3.44–5.89	10	4.03 (0.89)	2.56–5.30	13.50**
CLASS: Instructional support	18	3.26 (0.51)	2.40–4.33	10	2.71 (0.85)	1.53–4.13	5.71*
CLASS: Student engagement	18	5.15 (0.62)	3.67–6.00	10	4.48 (0.79)	3.00–5.67	3.55
Student self-reported outcomes							
Emotional engagement	190	3.95 (0.91)	1.00–5.00	34	3.46 (1.04)	1.00–5.00	7.97**
Behavioral engagement	190	4.27 (0.60)	2.14–5.00	34	3.98 (0.60)	2.57–5.00	6.75*
Cognitive engagement	190	4.01 (0.59)	2.25–5.00	34	3.83 (0.62)	2.67–5.00	2.44
CLASS—Student report—Emotional support	192	4.18 (0.61)	1.75–5.00	34	4.00 (0.72)	2.00–5.00	5.94*
CLASS—Student report—Instructional support	192	3.74 (0.60)	1.86–5.00	34	3.45 (0.73)	2.06–4.56	6.99**

** *p* ≤ 0.01,

* *p* < 0.05.

**TABLE 4 |:** Observed MTT implementation integrity during focus/plan and reflect/share sessions.

Focus/Plan session (*n* = 11)	Mean (SD)	Range
Facilitator introduces MTT topic	1.63 (0.50)	1.00–2.00
Participants review the MTT strategy^a^	0.45 (0.82)	0.00–2.00
Participants discuss strategy implementation	1.81 (0.60)	0.00–2.00
Facilitator prepares participants to analyze provided example video	1.25 (0.30)	1.00–2.00
Team views provided example video	1.63 (0.50)	1.00–2.00
Participants discuss observations/analysis #1	1.45 (0.93)	0.00–2.00
Participants discuss observations/analysis #2	1.27 (1.0)	0.00–2.00
Participants discuss observations/analysis #3	0.67 (1.15)	0.00–2.00
Facilitators orients team to planning	1.4 (0.70)	0.00–2.00
Participants make plans to implement MTT strategy	1.25 (0.88)	0.00–2.00
*Mean implementation integrity across all Focus/Plan sessions*	1.41 (0.31)	0.89–1.89
Reflect/Share sessions (*n* = 10):		
Facilitator orients to reflect/watch own video	0.70 (0.94)	0.00–2.00
Participants review own video and select segment to share before or during meeting	2.00 (00)	2.00–2.00
Facilitator reviews guidelines for sharing	1.5 (0.53)	1.00–2.00
Participants share video for feedback	2.00 (00)	2.00–2.00
*Mean implementation integrity across all Reflect/Share sessions*	1.52 (0.32)	1.25–2.00

All items rated on 3 point scale: 0 = not implemented, 1 = implemented with some omissions or errors, 2 = fully implemented as designed.

a Participants had reviewed strategies prior to sessions in 36% of sessions.

**TABLE 5 |:** Observed discussion topics and research supported collaborative practices for MTT and comparison group meetings.

	All MTT sessions (*n* = 21)	Focus/Plan MTT sessions (*n* = 11)	Reflect/Share MTT sessions (*n* = 10)	All comparison sessions (*n* = 4)	Total (*n* = 25)	MTT vs. Comparison		MTT Focus/Plan vs. Comparison		MTT Reflect/Share vs. Comparison	
	M (SD)	M (SD)	M (SD)	M (SD)	M (SD)	Mann Whitney U	*p*	Mann Whitney U	*p*	Mann Whitney U	*p*
**Observed discussion topics** ^a^											
Teaching practice	3.0 (0.0)	3.0 (0.0)	3.0 (0.0)	2.0 (0.00)	2.84 (0.37)	0.00	0.00*	0.00	0.001*	0.00	0.001*
Challenging student behavior	1.2 (0.75)	0.91 (0.70)	1.5 (0.70)	1.3 (0.50)	1.2 (0.71)	41.5	0.97	16.00	0.279	14.5	0.385
Challenging technology systems	0.95 (0.59)	0.82 (0.40)	1.1 (0.74)	1.3 (0.95)	1.00 (0.65)	32.00	0.49	16.00	0.236	17.5	0.705
Challenging school policies	0.48 (0.81)	0.64 (0.92)	0.30 (0.67)	1.0 (1.2)	0.56 (0.97)	31.00	0.45	19.00	0.477	13.0	0.212
Logistics/mechanics	0.81 (0.75)	0.73 (0.64)	0.90 (0.88)	3.0 (0.00)	1.16 (1.07)	0.00	0.00*	0.00	0.002*	0.00	0.004*
Other topics	1.1 (0.73)	1.2 (0.75)	1.1 (0.74)	0.75 (0.50)	1.08 (0.70)	29.00	0.37	18.00	0.429	14.5	0.384
**Observed research supported collaborative practices** ^b^											
Share ideas about teaching	2.00 (0.00)	2.00 (0.00)	2.00 (0.00)	2.00 (0.00)	2.00 (0.00)	42.00	1.00	24.0	1.00	22.0	1.00
Self-reflected on practices	1.85 (0.48)	1.81 (0.60)	1.90 (0.32)	1.50 (1.00)	1.80 (0.58)	35.00	0.64	20.0	0.40	19.5	0.64
Review video of team members’ teaching	0.90 (1.00)	0.00 (0.00)	1.9 (0.32)	0.00 (0.00)	0.76 (0.97)	22.00	0.15	24.0	1.0	0.00	0.001*
Provide feedback to peers	1.48 (0.87)	1.00 (1.00)	2.00 (0.00)	1.25 (0.96)	1.44 (0.87)	35.00	0.64	19.5	0.55	11.0	0.02*
Make plans for teaching practices	0.67 (0.86)	1.09 (0.83)	0.20 (0.63)	0.75 (96)	0.68 (0.85)	39.50	0.86	20	0.61	13.5	0.11
Discussed student data^c^	0.19 (0.40)	0.18 (0.60)	0.20 (0.42)	0.75 (0.96)	0.28 (0.61)	26.50	0.26	14.5	0.09	14.0	0.18
Discussed other PD^c^	0.00 (0.00)	0.00 (0.00)	0.00 (0.00)	0.00 (0.00)	0.00 (0.00)	42.00	1.00	24.0	1.0	22.0	1.00

a Items rated on a four-point scale: 0 = no time, 1 = cursory mention, 2 = significant but <50% of meeting, 3 = significant >50% of meeting.

b Items rated on a three-point scale: 0 = not observed 1 = moderate 2 = highly effective.

c Not part of MTT intervention.

* *p* < 0.05.

**TABLE 6 |:** Predicting observed classroom experiences with number of MTT sessions and topics discussed in sessions.

	Observed emotional support	Observed instructional support	Observed student engagement
	Beta (SE)	Beta (SE)	Beta (SE)
Number of MTT sessions before observations	0.39 (0.14)**	0.30 (0.12)	0.35 (0.13)+
Review video of team members’ teaching	0.53 (0.30)**	0.42 (0.27)*	0.46 (0.29)*
Provide feedback to peers	0.30 (0.24)	0.26 (0.21)	0.21 (0.23)
Discuss teaching practice	0.52 (0.28)**	0.39 (0.25)*	0.44 (0.27)*
Discuss logistics/mechanics	−0.56 (0.12)**	−0.44 (0.11)*	−0.48 (0.12)**

*n* = 28.

** *p* ≤ 0.01,

* *p* < 0.05,

+ *p* < 0.10.

**TABLE 7 |:** Predicting student reported classroom experiences with number of MTT sessions and topics discussed in sessions^a^.

	Behavioral engagement (*n* = 190)	Emotional engagement (*n* = 190)	Cognitive engagement (*n* = 190)	CLASS-student report emotional support (*n* = 192)	CLASS-student report instructional support (*n* = 192)
	Beta (SE)	Beta (SE)	Beta (SE)	Beta (SE)	Beta (SE)
Number of MTT sessions before observations	0.19 (0.02)*	0.18 (0.03)*	0.09 (0.02)	0.10 (0.05)	0.18 (0.05)*
Review video of team members’ teaching	0.22 (0.16)*	0.20 (0.26)*	0.15 (0.16)+	0.41 (0.06)***	0.75 (0.07)***
Provide feedback to peers	0.08 (0.11)	0.02 (0.17)	0.06 (0.11)	−0.28 (0.04)***	−0.04 (0.05)
Teaching practice	0.22 (0.15)*	0.19 (0.24)*	0.14 (0.15)	0.43 (0.06)***	0.81 (0.06)***
Logistics/mechanics	−0.28 (0.07)***	−0.25 (0.10)**	−0.19 (0.07)*	−0.37 (0.02)***	−0.73 (0.03)***

a Regressions with student self-report controlled for teacher gender and race/ethnicity.

*** *p* ≤ 0.001,

** *p* ≤ 0.01,

* *p* < 0.05,

+ *p* < 0.10.

## Data Availability

The original contributions presented in the study are included in the article. Further inquiries can be directed to the corresponding author.
